# Voltage- and Ca^2+^-dependent SV/TPC1 ion channel structure at the onset of opening

**DOI:** 10.1073/pnas.2200610119

**Published:** 2022-03-02

**Authors:** John M. Ward

**Affiliations:** ^a^Department of Plant and Microbial Biology, University of Minnesota Twin Cities, St. Paul, MN 55018

To understand the changes in conformation that occur in ion channels upon activation it is necessary to have structures representing both active and inactive conformations. Two new reports in PNAS now show structures of *Arabidopsis* TPC1 in the early stages of activation (1, 2). Obtaining protein crystal structures for *Arabidopsis* TPC1 was an especially important breakthrough because the channel was shown in the inactive conformation (3, 4), while most previous structures for depolarization-activated ion channels showed the channel proteins in an activated state due to the way that they are regulated by membrane potential. Depolarization-activated ion channels are normally closed at resting potentials and are activated by depolarization of the membrane potential to less-negative values. Protein crystal structures of membrane proteins are obtained under depolarized conditions with protein isolated in detergent micelles in which no membrane potential is present. The reason that the plant TPC1 structure was captured in the resting state is actually very complicated. TPC1 activation requires both elevated cytosolic Ca^2+^ and depolarization, and TPC1 is inhibited by a luminal (outside) Ca^2+^ binding site involving D454, which interacts with the voltage-sensing domain (VSD2). In the original protein crystal structures, the luminal Ca^2+^ binding site stabilized VSD2 in the resting state.

The slow vacuolar (SV/TPC1) ion channel was one of the first plant ion channels to be identified and studied using the patch-clamp technique (5) and is found in most if not all land plant cell vacuoles. The plant vacuole is a large intracellular organelle analogous to the lysosome in animal cells. The SV channel is a nonselective cation channel found in the vacuolar membrane; it is Ca^2+^-permeable (6) and is activated by depolarization with the slow kinetics that led to the name “slow vacuolar.” The discovery that the *AtTPC1* gene encodes SV channel activity (7) provided the first information about the structure of the channel. The SV/TPC1 channel consists of two tandem channel domains analogous to the Na_v_ channel structure. Most Na_v_ channels are homotetramers with 24 transmembrane domains. The bacterial sodium channel Na_v_Ab is a homotetramer composed of four identical subunits with six transmembrane segments each, and 24 total transmembrane domains (8). TPC1 contains 12 transmembrane spanning domains, two pore domains, two voltage sensing domains (VSD1 and VSD2), and two Ca^2+^-binding EF hand domains within the central cytosolic loop. The predicted protein structure suggested that the SV ion channel consists of a TPC1 dimer in the membrane, and this was confirmed by genetic evidence (9) and by the protein crystal structures (3, 4).

Activation of TPC1 involves the physical movement of VSD2. VSDs are composed of four helical membrane spans, one of which has positively charged amino acid residues that move in response to membrane potential changes. In TPC1, VSD1 does not contribute to voltage regulation of the channel (3, 4). VSD2 is responsible for voltage sensing and contains four arginine residues separated by helical turns within one transmembrane span. At resting potential, VSD2 is attracted electrostatically to the cytoplasmic side of the membrane, resulting in a closed channel, and during depolarization VSD2 moves outward, toward the lumen side of the membrane. This conformational change is linked to dilation of the central pore which then conducts ions. Understanding the details of these conformational changes is the focus of the two new reports on TPC1 structures (3, 4).

To study the activation of TPC1, both research groups took advantage of mutations in the inhibitory luminal Ca^2+^ binding site. Ye et al. (2) compare a structure for wild-type TPC1 (AtTPC1_WT_) obtained in the presence of 1 or 50 mM Ca^2+^ which represents the Ca^2+^-inhibited inactive form of the channel with a triple mutant (D240A/D454A/E528A), designated AtTPC1_ΔCai_, at the same calcium concentrations. At 1 mM Ca^2+^ the AtTPC1_WT_ structure showed a closed pore, with VSD2 in the resting conformation, and unoccupied EF hand domains. Surprisingly, the AtTPC1_WT_ structure at 50 mM Ca^2+^ was the same, indicating that the resting VDS2 prevented occupation of the EF hands (2). The important implication for the physiological function of SV/TPC1 is that depolarization of the membrane and the subsequent VSD2 conformation changes are a prerequisite to channel activation by cytoplasmic Ca^2+^. Structures for AtTPC1_ΔCai_ at both 1 and 50 mM Ca^2+^ were similar to each other. Both showed major conformational changes associated with VSD2 movement and Ca^2+^ binding to the EF hand domains (2). Removal of the luminal inhibitory Ca^2+^ binding site allowed VSD2 to adopt the active conformation. Dickinson et al. (1) used a similar approach to obtain structures for activating TPC1. They used the overactive AtTPC1, *fatty acid oxygenation up-regulated 2* (*fou2*) mutant containing a D454N point mutation in *AtTPC*1 (9). The D454N change is known to decrease luminal Ca^2+^ inhibition and shift the activation potential to more negative potentials and causes channel hyperactivity and phenotypes related to the stress hormone jasmonate (9, 10). In the absence of Ca^2+^, VSD2 was highly mobile and distinct states were resolved, one of which corresponded to the wild-type resting state. Therefore, in the *fou2* mutant, in the absence of a membrane potential VSD2 can fluctuate between resting state and an activated state. States II and III represent conformations of the channel as it activates. In state II, VSD2 has rotated and moved up by one helical turn, transferring one charge toward the outside of the channel. These conformation changes represent the onset of channel activation (1).

The detailed structures for SV/TPC1 in the onset of opening greatly enhance our understanding of the voltage-dependent ion channel activation mechanism and contribute to our ability to study the physiological function of SV/TPC1 and other voltage-dependent ion channels.

The detailed structural analysis of SV/TPC1 makes it one of the best-characterized ion channels. However, questions remain about the physiological function of the ion channel. Several studies indicate that SV/TPC1 channels function during signal transduction in plants and that the channels are otherwise normally closed. We know that the requirements for channel opening, elevated cytoplasmic Ca^2+^ and a depolarized membrane potential, are not resting conditions but are conditions that occur during signal transduction. In plants, *TPC1* has been shown to be required for systemic signal transduction in response to salt stress; in the at*tpc1* loss-of-function mutant the Ca^2+^ wave propagation rate was decreased by a factor of 25 (11). Loss of *TCP1* also is known to inhibit Ca^2+^-induced stomatal closing and ABA-dependent repression of germination (7). In the *fou2* mutant, wounding causes more synthesis of the stress hormone jasmonate compared to wild-type plants (9).

To understand TPC1 activation in plant cells, we need to know what conditions could lead to depolarization of the vacuole membrane, also known as the tonoplast. The membrane potential of the vacuole membrane is controlled differently than at the plasma membrane. Proton pumps (vacuolar H^+^-ATPase and H^+^-pumping pyrophosphatase) generate the membrane potential (cytoplasmic side negative) by pumping H^+^ into the vacuole lumen (Fig. 1*A*). However, the vacuolar membrane potential is largely dissipated by a high conductance for anions, mainly Cl^−^ and NO_3_^−^ (12–14). This results in a relatively low membrane polarization, approximately −10 to −30 mV, and allows for the large pH gradient to be generated across the vacuole membrane, on the order of 1.5 pH units in most cells. Potassium is accumulated in plant vacuoles via H^+^/K^+^ antiporters in the NHX and CHX families (13, 15). There is a large transmembrane Ca^2+^ gradient across the vacuole membrane, the cytoplasmic Ca^2+^ concentration in resting cells is around 0.1 to 0.2 µM and during signal transduction it can increase to around 2 µM. The Ca^2+^ concentration in the vacuolar lumen is in the range of 2 mM, generated by H^+^/Ca^2+^ antiporters in the CAX family and Ca^2+^-ATPases. Elevated cytoplasmic Ca^2+^ in the range of 1 µM was shown to activate K^+^-selective VK/TPK channels in the vacuole membrane (Fig. 1*B*) which are fairly voltage-independent and could allow K^+^ flux into the cytoplasm and depolarize the vacuolar membrane potential (6). Thus, VK/TPK channel activity could couple elevated Ca^2+^ and membrane depolarization, the two conditions required for SV/TPC1 activation.

**Fig. 1.**
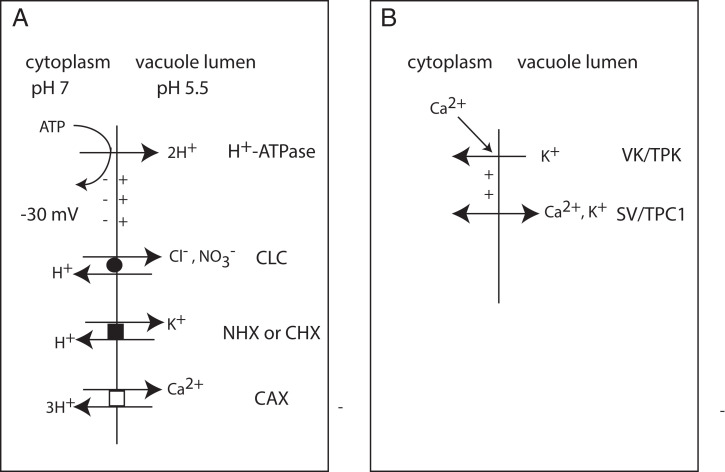
Transport processes at the vacuole membrane related to activation of SV/TPC1. (*A*) The negative membrane potential is generated by proton pumps; here only the V-type H^+^-ATPase is shown but the H^+^-pyrophosphatase also contributes. The membrane potential is mostly dissipated by Cl^−^ and NO_3_^−^ conductance of CLC channels, allowing the large transmembrane pH gradient. Potassium is accumulated in vacuoles by H^+^/K^+^ antiporters in the NHX and CHX family. Calcium is accumulated in the vacuole by H^+^/Ca^2+^ antiporters in the CAX family and Ca^2+^-ATPases (not shown). (*B*) SV/TPC1 activation requires membrane depolarization. VK/TPK channels are calcium-activated and selective for K^+^. Activation of VK/TPK channels would allow K^+^ movement into the cytoplasm and depolarize the vacuole membrane, activating SV/TPC1 channels which are nonselective Ca^2+^-permeable channels.

SV/TPC1 channels have a permeability ratio for Ca^2+^:K^+^ of 3:1 and show tail currents that carry Ca^2+^ flux into the cytoplasm, with an ~7-fold higher single channel conductance for K^+^ compared to Ca^2+^ (6), consistent with a higher Ca^2+^ affinity. Calcium permeability of SV channels has also been observed by combining patch clamp and fura-2 fluorescence (16) but only to measure Ca^2+^ flux into the vacuole. When SV channels conduct K^+^ in one direction, a fractional Ca^2+^ current (17) through SV channels was revealed in the opposite direction of K^+^ flux using this approach (16). A function for VK and SV channels working together to function in Ca^2+^-induced Ca^2+^ release from the vacuole (6) is a hypothesis. The detailed structures for SV/TPC1 in the onset of opening greatly enhance our understanding of the voltage-dependent ion channel activation mechanism and contribute to our ability to study the physiological function of SV/TPC1 and other voltage-dependent ion channels.

## Change History

March 21, 2022: Due to a production error, Fig. 1 appeared incorrectly. Fig. 1 has been updated.
